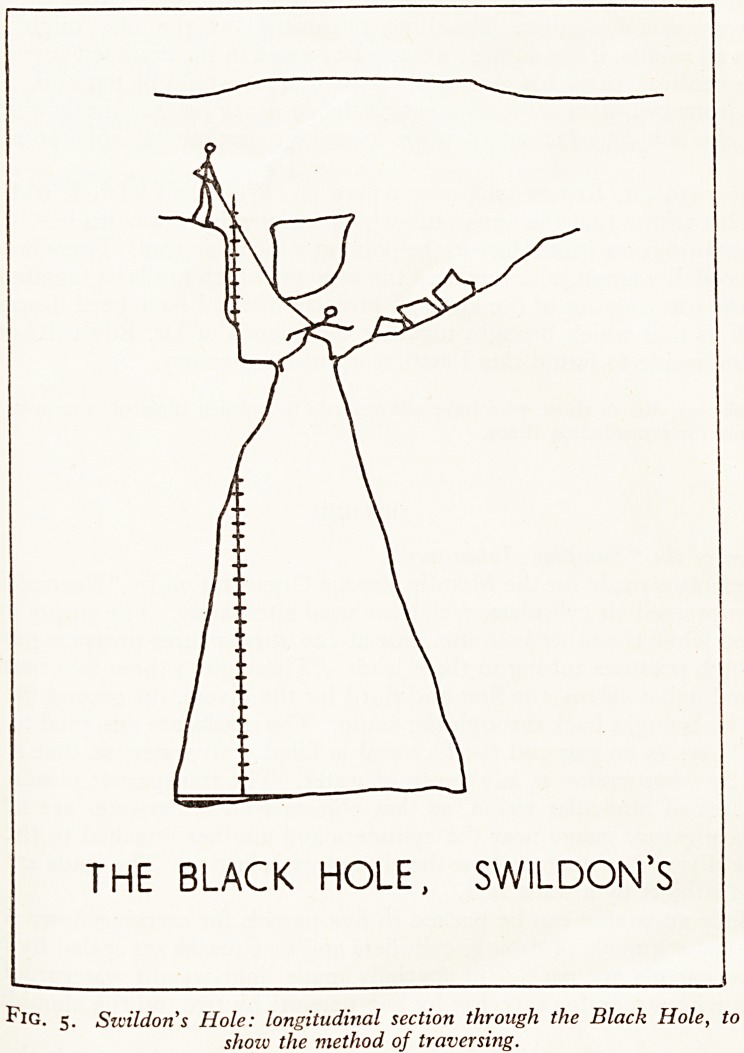# Cave Rescue

**Published:** 1961-04

**Authors:** Oliver C. Lloyd


### CAVE RESCUE

BY

DR. OLIVER C. LLOYD

*Edward Long Fox Memorial Lecture* 1960

The primary object of this lecture is to keep green the memory of Dr. Edward Long Fox. But who was Dr. Long Fox, and why should we remember him? He was a good physician but not a specially distinguished one. He wrote no classics and there is no disease named after him. But he was liked—well liked. He was the kind of man they chose to have as Chairman of this or President of that. And when he died in 1902 his friends founded this Lectureship in his honour. They were honouring a friend. The cement which unites members of the medical profession into one fellowship was the thing which guided them; and it can have been no accident that the title of the first of these Lectures, by Dr. John Beddoes, should have been "The Ideal Physician".

We have no record of the discussions which the founders must have had, when they were deciding the terms of the lectureship, so we don't really know what these lectures were supposed to accomplish. They were to be to a mixed medical and non-medical audience, which rules out anything too rarefied, and the scope given in the terms of reference is a wide one, namely "a subject connected with medicine or one of the allied sciences". One eminent descendent of Dr. Long Fox has suggested that this is wide enough to cover "The Secret of Successful Shoe-fitting". At any rate its liberal interpretation has given us the pleasure of admiring Stubbs' wonderful drawings of horses, shown to us not long ago by the Professor of Veterinary Anatomy. I have chosen as the subject of my lecture "Cave Rescue", because it has intrinsic interest and because it brushes gently against the fringes of medicine.

Not twelve miles from here lies the range of hills called "Mendip". These hills are full of caves, and their exploration is becoming an increasingly popular pastime among young people. As in the cognate sport of "mountaineering", which may be described as "caving in reverse", it is dangerous, a fact which may repel some people but certainly attracts others. It is hardly surprising therefore that cavers sometimes get into difficulties or get hurt, and the Mendip Rescue Organization exists for the purpose of helping cavers out of any kind of trouble which is too much for them to overcome on their own.

The records of this Organization go back for ten years. During this time there have been thirty call-outs, of which seven proved to be false alarms. These false alarms were mostly due to inaccurate information, or even to no information at all. Once, when some Wells boys were reported missing, the Rescue Warden found that they had already gone to bed. Another time a pair of shoes was found outside Stoke Lane Swallet, and the wardens had to make sure that the owner was not in the cave. On yet another occasion, though this was more than ten years ago, a set of feminine underwear was found outside G.B. Cave. I forget what was the explanation of this!

Many false alarms could have been prevented by the observation of one simple rule: all cavers when starting on an expedition should leave a message with someone on the surface to say where they are going and at what time they expect to return. Sometimes however, the party may not know which cave they are visiting, as in 1952, when a party of two set out for Rod's Pot and went down Drunkard's Hole by mistake. The fatter member of the party got trapped by a moving boulder, but the thinner was able to get out and raise the alarm.

Three call-outs have been made because of failed lights. This must be classed as an avoidable accident. It doesn't really matter what lights you use; even candles are adequate on short trips, provided you take dry matches with you in a waterproof container. But whatever you use, whether candles, carbide, or electricity, you must make sure before starting that all is in good order and that you carry with you the necessary spares.

Getting lost in a cave is an avoidable accident and has resulted in three call-outs. Each time the party was an inexperienced one. One party was in the boulder ruckle in Eastwater in 1955. Another was at the foot of Jacob's Ladder in Swildon's Hole in 1953 (from which arises one of my favourite aphorisms: "When in doubt, look up"). But the one with which I have the most sympathy was a party of R.A.F. apprentices, who got lost in the boulder ruckle under the main chamber of Read's Cavern in 1953. When you are in solid rock it is fairly easy to get your bearings by the dip and strike of the rocks. But in a boulder maze you get no such help. I was nearly lost in the same place, once, and it was only because I had marked the turnings that I found

FIG. 1. *Eastwater cavern; the Primrose Squeeze. Longitudinal section, from a survey by D. Warburton, published by the Wessex Cave Club.* 1954.

my way out. Some people unroll a ball of twine in the Eastwater maze, like Theseus in the Palace of Minos; but this is generally considered unsporting.

### IMPACTION IN A "SQUEEZE"

There were three cases of people getting stuck in very narrow places; and these too, I'm afraid, must be classed as avoidable. If it is axiomatic that you don't climb down something you can't climb out of, then by the same logic you don't force yourself into a squeeze that you cannot wriggle out of. But it is not quite as simple as that. If the return through the squeeze is delayed, then cold, exhaustion or lowered morale may make impossible something that was only difficult.

A case in point is the incident in the Primrose Squeeze in Eastwater in 1958 (Fig. 1 and Plate V). This formidable squeeze is 20 ft. long, is inclined at an angle of 30 degrees and has at its lower end a pot hole 185 ft. deep, of which the first section is of 32 ft., down onto a ledge. Personally I can't get through the squeeze; at least I don't think I can, and if anybody were badly hurt on the far side, I really don't know how we should get him out. But that is not what happened. Laddering the Primrose Pot is a tedious business, and if you want to get two or three people to the bottom and remove the ladders all in one trip, it will take twelve hours. So this time the laddering was done by one party the previous day, and then a second party went down, of whom it was thought that only two could get through the squeeze. One failed and the other, a beginner, succeeded. He took two and a half hours getting to the bottom of the 185 ft. ladder pitches, another hour getting up again, and by that time he was so exhausted that he couldn't get back through the squeeze. He was tired, he was hungry, and he had been far too long on the wrong side of that squeeze. You may call this bad generalship; it wasn't his fault. So they sent for help, which came in the form of a small caver, who could get through the squeeze, give the exhausted beginner something to eat and encourage him from behind. In this he was successful, and the subject returned through the squeeze under his own steam.

It is usually possible to "talk" someone through a squeeze: a form of morale boosting. And it may also be said that if a subject cannot get himself back through a tight place and gets stuck, then nothing will move him. In the case of the Peak Disaster, to which I shall refer, the subject could have been talked out of that squeeze, if he had not been overcome by foul air.

Moving boulders accounted for three call-outs in the period under review. It is not always an avoidable accident. The circumstances under which a lad was crushed last year by a falling boulder in Dow Cave, up in Yorkshire, were probably unavoidable. Rock falls, sometimes of considerable size, are known to occur even when there is no one about, as in the Dolphin Vertical twelve months ago. This used to be a very much frequented ladder pitch, and it gave us the cold shivers, when we saw what had happened. The avoidable accidents usually occur on boulders which are frequently passed, where familiarity brings with it a certain lack of respect. A classic example is that which occurred in G.B. Cavern in the ox-bow in 1952. Two cavers were climbing up this boulder slope, as they had often done before, and the front man dislodged a large rock, which rolled down, bounced and caught the second man by the ankle against the wall. It took eighteen cavers and a strong rope to release him. It was a long time before he went caving again.

### EXHAUSTION

Exhaustion is another avoidable accident. It has a mixed origin. Usually the subject is inexperienced, temperamentally unsuited to caving, and has not had enough to eat. When such a person gets cold and tired he may go into a state akin to surgical shock, with prostration, slow cerebration and a thin rapid pulse. His reactions become slow, he doesn't respond to commands and he just seems to want to curl up. The condition is dangerous and has resulted in one death on Mendip and probably two others in the North. Most of the Mendip cases occurred in Swildon's Hole.

One which took place in 1956 was particularly instructive. The girl was one of a mixed party of six, who went down the cave one night without having a proper meal beforehand. It was her first caving trip. Now Swildon's Hole to the first sump or siphon is a distance of 1900 ft., and the sump is 380 ft. below the entrance. The stream passage is one of exceptional beauty and for those who like that sort of thing it is a continual delight. To negotiate it one has to descend a pothole 40 ft. deep, and soon after that another one of 20 ft. (Plate VI). This one looks pretty wet, but I can assure you that "the forty" is even wetter as a rule. A little further come the Double Pots (Plate VII), with their folded rocks and stalactite decorations. Old Balch used to say that no one could be called a true son of Mendip that hadn't fallen into the Double Pots. Passing below Tratman's Temple (Plate VIII), with its rows of delicate stalactites and columns, the stream passage (Plate IX) appears more forbidding, and when one reaches the First Sump (Plate X) it is time for most people to turn back.

FIG. 2. *Swildon's Hole: longitudinal section through the 40 ft. pot and the water rift above it, to illustrate the technique of hauling up a subject. Horizontal lines indicate pools of water; the waterfall coming from the iron pipe is indicated by three broken lines. The life-liners are standing on rock just behind the iron pipe.*

This girl felt tired after getting down the ladder pitches, but was assured by her companions that she would be all right, and the party continued to the Sump, where she collapsed with exhaustion. It was only then that they appreciated the seriousness of the position and began to assist her back to the surface. It was with difficulty that she could climb the 20 ft. pot and "the forty" became an impassable barrier. By this time the morale of the whole party was shaken and two of them left the cave to get help.

Their progress was not assisted by having one of the wires of their ladder break under them, a fact which they neglected to report to the Rescue Organization. There is outside of each of the principal caves on Mendip a notice giving the name of the cave, the telephone number of the Wells Police and the situation of the nearest telephone. They read the notice and followed the correct procedure, which is so much more satisfactory than taking short cuts, that I will digress a moment to describe it.

The alarm is given to the Wells Police: Wells, because it is conveniently placed, and the Police, because generally speaking any type of rescue work is their business. Cave rescue is our business, because it is a job that cavers can do and nobody else can. The relationship therefore of the cave rescue organization to the Police is a partnership. We look upon them as valuable allies and they have the same regard for us.

The Police need to know as much as possible about the accident, particularly where it is, what it is, and where the call is being made from. This last is because the caller is required to remain by the telephone until the Rescue Warden rings him back. The Police ring up the Rescue Wardens in list order until they find one (they always manage to find one), and together they decide what is to be done. The Warden then rings back the caller who gave the alarm, gets the rest of the news from him and tells him what to do. In all cases the Warden contacts the Medical Warden or one of his Deputies, so that there is always a doctor at hand in case of need. The need may not be obvious in the first place, but may arise quite suddenly, particularly in cases like the one I was describing, and to which I will now return.

The rescue party consisted of five cavers and the Medical Warden. When they found the girl she was cold, miserable and trembling violently. Three important restoratives were then given her: glucose by mouth, to correct the hypoglycaemia, dexedrine as a stimulant, and encouraging conversation. These did much to restore her spirits. She was then wrapped in the carrying sheet and hauled up the 40 ft. pot, after which she was able to go most of the way out by her own efforts.

Hauling a subject up "the forty" presents certain problems. In this case three people were easily able to raise the subject who weighed only 9 stone by direct pull, but the rope kept jamming in the crack by the iron pipe. A fourth caver on a separate line climbed the ladder by her side and often had to support her entire weight while the hauling line was being freed.

We carefully considered this problem afterwards and solved it by fixing an iron bar across the rift at the top of the pot, known as Suicide's Leap (Fig. 2). To this a pulley may be attached, and on subsequent practices and on one rescue also we have shown that it is easy to haul a subject up the pitch without any jamming. On one of these practices the subject weighed 16 stone and we needed six men on the hauling line. Two life liners stood at the head of the pitch, one to hold the subject and the other to hold the man climbing the ladder. On reaching the top the man on the ladder passed to the life liners a short line tied to the subject's feet. The subject could then be easily pulled feet first onto the platform, while the haulers let him down.

The restoratives to which I referred may sometimes be supplemented by physical exercise. There was an occasion in 1958 when two parties went down Swildon's Hole, using the same ladders, but without making proper arrangements about who was going where, or who was likely to be the last out. As a result party A came out of the cave, removing all the ladders, and then discovered that party B was still down. Well, they called out Mendip Rescue, and a party went down the cave about an hour and a quarter later, meeting the lost ones at the bottom of the 20 ft. pot. There were two youths and two girls. One of the girls was on her first caving trip and was in poor condition, shivering violently and slow to respond to instructions. They were given glucose and all climbed "the twenty" satisfactorily except this girl, who lost her helmet, lamp, and one shoe on the way up. The Rescue Warden then got one of the chaps to walk her up and down until they were ready to go back to "the forty". The result was that she had perked up quite a lot by the time they reached it and did not find the climb unduly difficult.

### INJURIES

Severe injuries down caves are not very common. We have had six cases in ten years. Two of these have been falls due to an improper use of the life line. In one case no life line was used at all, and a beginner was allowed to climb a slippery 50 ft. ladder in Rod's Pot. She fell and broke her leg. In another case the subject did not tie a proper knot in his life line and fell down the Second Vertical in Eastwater, a distance of 40 ft., and sustained head injuries with fractured clavicle and ribs. He was unconscious for all of the twelve hours we took to get him out of the cave. It is absolutely essential for cavers to be able to tie a bowline. They should be able to tie it with one hand and with their eyes shut.

It is important to begin moving an injured subject out of a cave as soon as possible. First, because no definitive treatment of the injury can be done until he is outside, second because delay in beginning definitive treatment may be fatal, and third, because it helps to sustain his morale. To this end a certain minimum of first aid should be known by the rescuers. They should know how to move the subject. They should be able to recognize the deformity of a fracture and know how to immobilize the limb. If the back has been broken the subject should be carried face downwards in the carrying sheet. This keeps the spine in extension and prevents any contusion of the spinal cord from being made worse. The value of this treatment was demonstrated at the Eastwater rescue last July.

But on Mendip we rely rather more on medical aid. We are singularly fortunate in having a number of medically qualified cavers. When I was at a conference of cave rescue organizations in Settle last year, I was surprised to hear the caving doctor described as a "specialist", along with the diving and explosives experts. In the North they have great difficulty in finding doctors willing to go down caves or able to do anything effective when they get down them. But on Mendip they are two a penny. This is because of the close association there has always been between the Medical School here and the University Spelaeological Society. The Society was run by medicos for some decades. The Professor of Pathology was at one time Secretary of the Society, and our present Pro-Vice-Chancellor was for many years its Treasurer. No such association exists between a caving organization and any other medical school in the country; but then no other medical school is so fortunate as to have such a good caving area as Mendip on its doorstep.

So when last year a lad of 17 fell down the Coal Shute in Goatchurch Cavern and broke his leg, Dr. Crook went down with the rescue party and gave assistance which could not possibly be described as merely first aid. The fracture was of the tibia and fibula but the subject was in good condition. Dr. Crook removed the boot and cut up the overalls, but left the sock and trouser leg in position. Getting others to apply the necessary extension he then put the leg in plaster over the sock and trouser. After about 15 minutes the plaster had set and the subject was able to crawl out of the cave on his hands and knees, with one man supporting the plaster, and with no pain. This form of emergency treatment for a cave injury is revolutionary. It is far better than morphia. It has the great advantage of helping the subject to help himself. In this way he keeps warm and his morale is sustained. He should always be made to do as much for himself as possible. The drawbacks to this form of treatment are that it can only be done by someone familiar with plaster technique. The plaster must be padded (in this case by sock and trouser) but must be firm enough to prevent movement and consequent pain. The only people who complained in this case were the Casualty Officers at the B.R.I., who found that the plaster could not be cut with shears, and had to take the boy up to the theatre and use a circular saw.

PLATE V

Photograph by D. Warburton.

*Eastwater cavern; a caver coming up through the Primrose Squeeze.*

PLATE VI

Photograph by Dr. D. M. M. Thomson, 16/11/52.

*Swildon's Hole: The 20 ft. pot.*

PLATE VII

Photograph by Dr. D. M. M. Thomson, 16/11/52.

*Swildon's Hole: stalactites and folded rock over the Double Pots.*

PLATE VIII

Photograph by Dr. D. M. M. Thomson, 1/3/53.

*Swildon's Hole: Tratman's Temple.*

PLATE IX

Photograph by Dr. O. C. Lloyd, 5/1954.

*Swildon's Hole: Main Streamway below Tratman's Temple.*

PLATE X

Photograph by Dr. D. M. M. Thomson.

*Swildon's Hole: the First Sump.*

PLATE XI

Photograph by A. J. Morrison, April, 1957

*Swildon's Hole: crossing the Black Hole Traverse.*

PLATE XII

Photograph by A. J. Morrison, 10/1/60

*Sumping Apparatus: practice in a swimming bath. Subject being let down into the water, ready to be taken back through the sump. The face masks are well shown.*

PLATE XIII

Photograph by A. J. Morrison, 10/1/60

*Sumping Apparatus: practice in a swimming bath. Shows distribution of air lines to the two divers and to the subject, and the life line attached to the subject's legs.*

PLATE XIV

Photograph by A. J. Morrison, 10/1/60

*Sumping Apparatus: practice in a swimming bath. The full-scale model is seen under water in the shallow end of the bath; divers are passing through it.*

UNEXPECTED FLOODS

The danger of flooding in caves has probably been exaggerated, but this hazard does account for five operations by Mendip Rescue. The first of these was in 1951 at the Ogof Ffynnon Ddu in South Wales, but this was not really our show, though our party did have the distinction of being the first to make contact with those underground.

The other four occasions were all in the last two years. The first was at Swildon's Hole in January 1959, when persistent drizzle at mid-day over frozen ground caused the stream to flow over the entrance grating and this made the ascent of the 40 ft. pot almost impossible. There was a party of forty cavers underground on a diving operation in Swildon's IV, which is a particularly remote part of the cave. It was exceedingly difficult for the Rescue Organization to make contact with them below the 40 ft. pot, and between the hours of 6.0 and 9.30 p.m., when most of them came up, the flood water was still at a dangerous level and there was one fatality due to cold and exposure. I think a lot of lessons were learnt on that occasion. On the part of the rescuers, never to undertake that kind of operation without full equipment to deal with emergencies, or plenty of helpers. For the trapped party, not to "press on regardless". There are good reasons for not trying to climb "the forty" during flood conditions. A strong man can do it against the force of the water, but his weaker companion may not be able to. In either case there is the additional hazard of being hit by boulders carried down by the flood.

The second occasion was at St. Cuthbert's Swallet a year later, this time caused by heavy rain. A not very strong party of ten was down the cave and some found it impossible to climb the entrance pitch against the force of the water. This 25 ft. pitch is narrow and difficult at all times, but is kept dry by damming the stream near the entrance. Under flood conditions the dam arrangement breaks down. This operation took about 6 hours and was only made possible by getting the Fire Brigade to pump the water out of the dam pool. During these hours the rescue party successfully coped with the dangers of cold, exhaustion, and despondency to those trapped below. Two rescuers went down with food and hot drinks and they were supplied with exposure suits belonging to Mendip Rescue. But one factor contributed even more than the food and protective clothing to raise the morale of the trapped party and that was the fact that one of the rescuers was a woman.

The last two occasions were on the same day in August of this year and in the same two caves, but as a result of these previous experiences the Rescue Organization was able to cope with both emergencies at once. At St. Cuthbert's the party trapped was small, but the fire pump again helped to solve the problem. At Swildon's the flooding following a cloudburst was very severe. At 3 p.m., when the last party entered, the water was rising but not yet up to the grating. At 4.0 p.m. the water was 2 ft. deep over the grating and one couldn't see where the entrance was. The rain then stopped and the waters receded at first rapidly and then more slowly. There were eleven cavers below the 40 ft. pot and eleven more in Upper Swildon's. We knew of course, that when water flows over the entrance grating the 40 ft. pot is impassable, and that we should have to do something as soon as possible to relieve those having to wait down below. But we were not particularly anxious about those in Upper Swildon's, as there are plenty of nice dry safe places there, where one can wait.

In this we were wrong. At the height of the flood a party of four was in the Water Rift between the first and second Stalagmite Barriers, returning from looking at the head of "the forty". One of them just managed to get through the first barrier, when it sumped and the other three were trapped. They saw the water rising at the rate of about 3 ft. in 3 minutes to a depth of 13 ft. in a rift only 16 ft. high, a level which it maintained for about half an hour before subsiding. If the rain had been much heavier or more prolonged these three would have been drowned. At times the water here is known to flood to the roof; you can see bits of straw up there, stuck in the cracks. (If you want to know which parts of a cave get flooded, go and have a look at it just after a heavy storm and study the distribution of foam and flood debris.)

We waited at the entrance until the water was only three inches above the grating and then sent down a party to fix the hauling gear over the 40 ft. pot. As it happened nobody in the end needed to be hauled up, but it was possible to send rescuers down by this means considerably before the ladder became climbable. They took with them soup and cooking materials, and made contact with the trapped parties at about midnight. The latter were wet and cold after their long wait, but there was only one casualty among them. A relatively inexperienced caver tried to climb the Double Pots, was swept off his feet and carried downstream for about 200 ft. He was found much later by one of the other parties, in the dark, and with no shoes or helmet, just above Barnes' Loop. (He has the satisfaction of knowing that, according to Balch's definition, he is now a "true son of Mendip", having fallen into the Double Pots.)

The Rescue Organization established telephonic communication down "the forty", and between the head of that pitch and the entrance. The Police provided us with walkie-talkie apparatus, which gave us effective communication with our base on Priddy Green. Spare boots, helmets and underclothes (besides exposure suits) were sent down to the trapped parties, since a few of them were not properly equipped for such an emergency. I've often wondered whether the owners got their spare clothes back.

At 12.45 a.m. the Bristol Waterworks started up their pumps a quarter of a mile up stream, removing water from the stream at 12,000 gallons an hour, and soon afterwards the Fire Brigade, having finished work at St. Cuthbert's, came and removed water at the rate of 24,000 gallons an hour with an incredibly efficient little portable pump. This soon dried up the stream completely and the trapped parties were able to climb back up "the forty". They were all out by 4.0 a.m. There is one interesting hazard to be noted with the use of a fire engine. If you have it too close to the entrance of the cave the draught may carry the exhaust fumes down the cave and make the atmosphere very uncomfortable for those below. When we found that was likely to happen, we moved the pump.

It would appear that the victims of this Swildon's flood have come in for a certain amount of blame, mainly from people who have never been down a cave, or who have long ceased doing so. It is true that emergencies of this sort do show up defects of organization. These may be inadequate equipment or bad leadership, or other sins which cavers have no business to commit, and which in favourable circumstances might pass unnoticed. But the behaviour of the water in Swildon's is difficult to predict. I have studied it fairly closely. It will take any amount of moderate continuous rain. It is the freak storm, coming suddenly, that floods it. We know now that, when a party is known to be below the 40 ft. pot, and water flows over the entrance grating the Rescue Organization must be called out. A party trapped in this way should wait in a safe, dry place (such as Barnes' Loop or Tratman's Temple) until rescued.

### FOUL AIR

I now come to the problem of foul air in caves. At the time of the Peak Cavern disaster in March 1959, when Neil Moss lost his life, nobody had ever seriously considered foul air as a caving hazard, so that it was a long time before anybody realized what was the matter. In caves the air is nearly always fresh, and only in the smaller dead-ends is there no draught at all.

The circumstances of the Neil Moss incident were these. He was exploring at the bottom of a 40 ft. tube with a cross section of about 2½ ft. by 1 ft. (Fig. 3). He had descended on a ladder, found a boulder choke at the bottom, and kicked the boulders around a bit to see if he could make a way on, thereby effectively jamming the lower end of the ladder. When after about half an hour he came to re-ascend the ladder, he found that he couldn't raise one foot above the other. He felt beaten after a further quarter of an hour or so and asked to be pulled up. He tied the end of a rope round his body, but the effect of six people pulling on a rope going round several sharp corners was merely to break the rope. This happened twice, during which time he made about 2 ft. of progress but then got firmly stuck. This must have effectively cut off all circulation of air, if indeed there had been any before. He became light-headed and then unconscious within about 2 hours, breathing stertorously, and after about another 24 hours was dead.

FIG. 3. *The Peak Cavern Disaster; longitudinal section to show the tube in which Neil Moss got stuck; modified from a drawing by L. B. Salmon in* Cave Science, *May* 1960, *Vol.* 4, *No.* 30.

Even when he became unconscious a quite incorrect appreciation of his trouble was made by the rescuers, and for hours large quantities of oxygen were brought in and piped down to him, with no real effect whatever. This is one of the respects in which the north country is at a disadvantage through having so few medical cavers. For even a student, provided he had studied his physiology attentively and had done his anaesthetics course, could have told them that it was not oxygen lack that Moss was suffering from, but carbon dioxide excess, which is far more of a menace.

Carbon dioxide excess can only be relieved by changing the air. The apparatus necessary for doing this was made some time ago for the Navy. It consists of a Venturi system—a sort of giant suction pump. Dr. Allan Rogers has shown that, if the nozzle of this is bored out with a No. 77 drill (0·018 in. dia.), a flow of 5 litres of oxygen per minute can cause 75 litres of air to be sucked through the system. The air is passed over soda lime in a canister, where the carbon dioxide is absorbed, and released in a purified state. This apparatus was not available at Peak until after Moss was dead. The time limits between which it is likely to be effective are fairly narrow, which makes its availability all the more urgent. If the rescuers had appreciated his trouble at the earliest possible moment, and if this apparatus had been available at the Derbyshire rescue organization's headquarters, it is still anybody's guess whether Moss could have been saved. But it is quite certain that he could be saved only by his own efforts, and for that he needed clean air.

You may say that the Peak Disaster is unique; that such a combination of circumstances could never happen again. I'm not so sure. I know of two or three places in Mendip caves where candles have a tendency to burn sluggishly. The most striking of these is in the Baker Series of the Banwell Bone Cave, where in certain seasons the air will not support a candle flame and the wood of a match will not burn. This means that there is a deficiency of oxygen, which is inconvenient but not necessarily lethal. What we don't know is whether there is also an excess of carbon dioxide there, and that is one of the things we mean to find out.

### RESCUE THROUGH A SUMP

Another problem is how to get an incapacitated subject back through an underwater trap, or "sump" as we call it. This problem, I am glad to say, has been solved.

FIG. 4. *Swildon's Hole: longitudinal section through the First Sump. The arrow indicates the direction in which the stream is flowing.*

There are on Mendip five sumps which can be free-dived, that is to say, which can be passed without the use of any breathing apparatus. Only two of these are commonly used by cavers. The one in Stoke Lane Swallet is only two feet long in normal conditions, but extends indefinitely in times of flood. The other in Swildon's Hole is $6\frac{1}{2}$ ft. long (Fig. 4). Beyond Swildon's First Sump lies an extensive series of passages, including the streamway and the Black Hole Series. The latter takes its name from a yawning chasm 40 ft. deep, approached from near the top (Fig. 5). The way on to further passages is by a traverse, where one has to step across a four foot gap with 30 ft. of nothing beneath one (Plate XI). It will be appreciated that this gives ample opportunity for accidents, but so far, I'm glad to say, none has occurred.

FIG. 5. *Swildon's Hole: longitudinal section through the Black Hole, to show the method of traversing.*

The earliest attempts to devise some means of bringing an injured man back through the sump resulted in the design of a simple face mask and re-breathing bag, the idea being that the subject could probably re-breath his own expired air safely for one minute, without getting acutely uncomfortable. Fortunately this was never tried out in practice.

The second idea, which we started three years ago, was to use an Amphibious Tank Escape Apparatus. This consists of a re-breathing bag which can be filled with oxygen, a small soda-lime canister to absorb carbon dioxide, and a tube held in the mouth by a gag. The oxygen in the bag lasts 4½ minutes in water at 65 deg. F and probably much less in water at cave temperature, which is 51 deg. F, because one breaths more deeply and rapidly in cold water. I tried this one out in Swildon's sump. It is tricky to use. A nose clip is essential. The gag is rather easily lost; and it is very buoyant. I concluded that it would be very difficult to use it on an injured man who had never held a breathing tube by a gag in his mouth before, let alone worn a nose clip.

Then along came doctors Oliver Wells and Allan Rogers fresh from the Peak Cavern incident last year. The former concluded that the problems of foul air and water as hazards were not dissimilar. Breathing apparatus for the one might do for the other. At all events, if the subject were to be moved in the cramped surroundings of a sump, he couldn't carry his air supply with him; it would be too bulky. It must be supplied from cylinders through a long tube to a gag or face mask which could be worn by the subject. He set to work to make experimental apparatus along these lines.

In June 1959 Dr. Rogers took over where Dr. Wells had left off, and it is largely owing to his efforts that the apparatus which we now have was made.*

And that brings me quite close to the point at which I started. There is a very strong feeling of sodality amongst cavers, and the cement, which binds us together and makes possible the carrying out of the kinds of projects which I have been describing, is the same stuff as that which brought together the friends of Dr. Edward Long Fox and made them decide to found this Lectureship in his memory.

My thanks are due to those who have allowed me to publish their photographs, and to Mr. D. N. White for reproducing them.

### APPENDIX

*Description of the "Sumping Apparatus"*

The apparatus made for the Mendip Rescue Organization by "Normalair" consists of two compressed air cylinders, which are used alternately. The empty cylinder may be changed while the other is in use. Air at 120 atmospheres pressure passes through 60 ft. of high pressure tubing to three leads. These go to three face masks with on-demand reduction valves, the first and third for the divers, the second for the subject who is to be brought back through the sump. The masks are designed to fit any type of face. There is no gag and the face seal is filled with water, so that it will not be distorted by compression at any depth of water. The transparent plastic vizor is flat over the area of binocular vision, so that objects seen underwater are not distorted. There is a pressure gauge near the cylinders and another attached to the third lead, so that the divers also may see how the air supply is lasting. The leads are attached to divers and subject by a waist belt.

The whole apparatus can be packed in five parcels for carrying down a cave. The joins between segments of tubing, cylinders and face masks are sealed by blanking-off nuts. The parcels are packed in specially made light-weight waterproof fibre-glass containers with straps for carrying by one person; by two, on the shoulder or in the hand.

The apparatus was first tried out in January, 1960, in a swimming bath, using a full scale model of Swildon's first sump (Plates XII, XIII and XIV). Both the divers and the subject need about 25 lb. of lead weights each, worn on a belt to correct for

* The film "Cave Rescue" was then shown. This film was made by members of the Mendip Rescue Organization, and the photography was by Mr. A. J. Morrison. A summary of the Lecturer's commentary is given in the Appendix—Ed.

buoyancy. The subject should be a little heavier than water, so as not to scrape against the roof of the sump. He is wrapped in a simple St. John's Ambulance type carrying sheet, laced up in front by means of cords from the feet towards the head. This allows for last-minute adjustments. A life-line taken through the sump by the divers is attached to his feet, as he will be drawn gently back through the sump feet foremost (Plate XIII). In this way his motion can be most easily controlled by the divers, and the second diver can see that he is in no distress. Care is needed in adjusting the mask on the subject. The back-plate should be drawn well down the back of the head before tightening the three pairs of straps. The pair behind the chin must not be drawn too tight, or respiration may be obstructed.

Telephonic communication is established between the two sides of the sump, and those on the near side are told when the return journey is about to be made. They then draw in the life line attached to the subject and the high pressure air tubing, so that no coils are left lying about in the sump.

The second practice was carried out in Swildon's Hole in May, 1960, at the first sump, and this too was illustrated in the film, which concluded by showing the technique of hauling a subject up the forty-foot pot in Swildon's Hole.

## Figures and Tables

**Fig. 1. f1:**
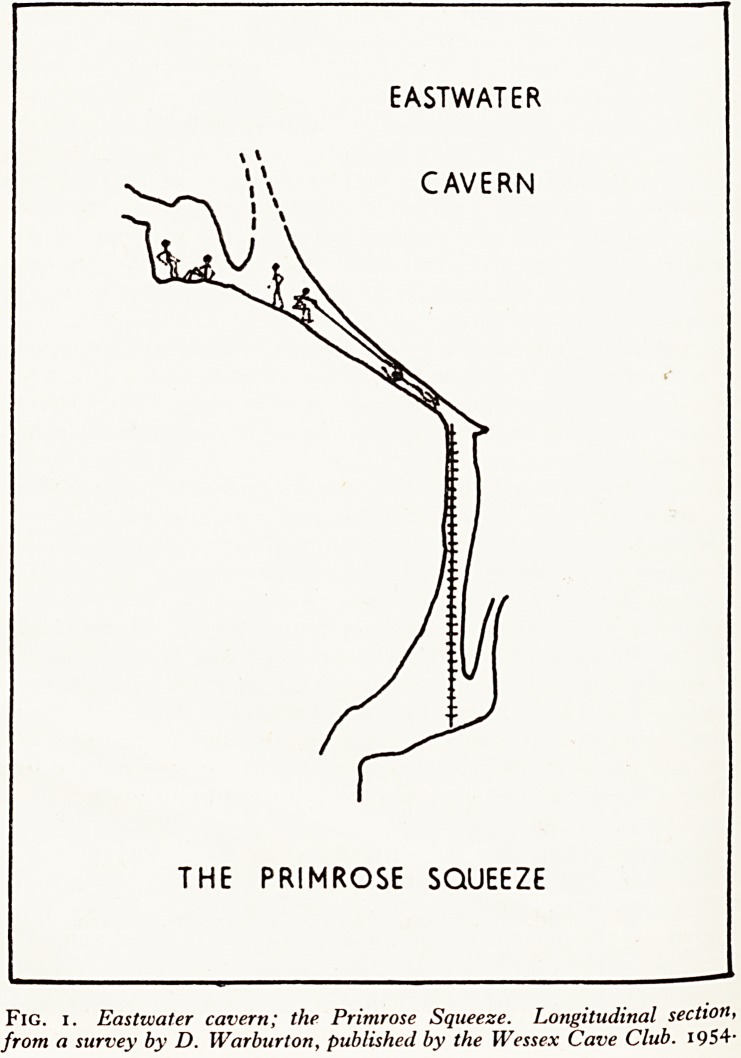


**Fig. 2. f2:**
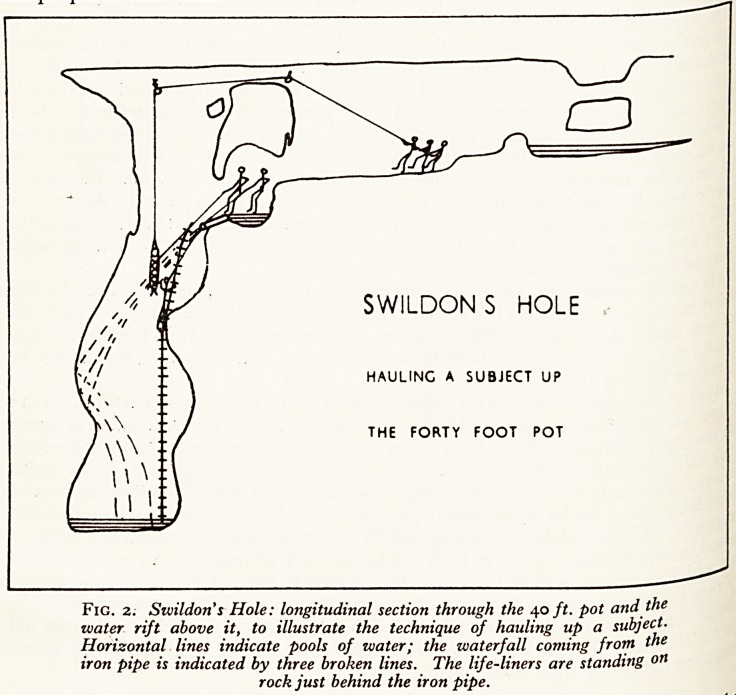


**Figure f3:**
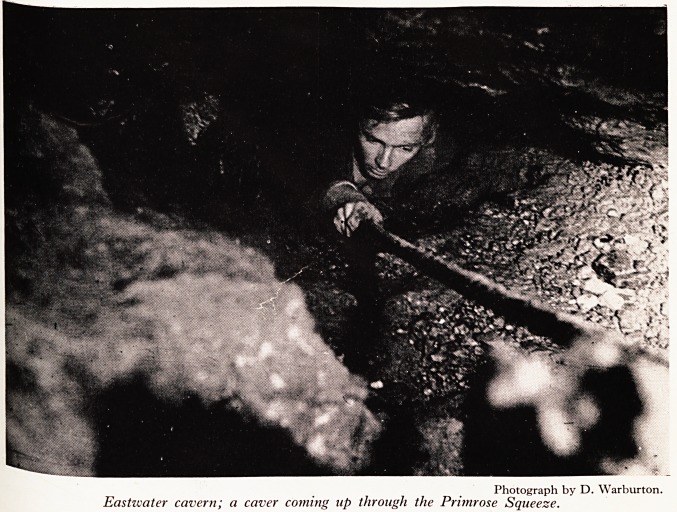


**PLATE VI f4:**
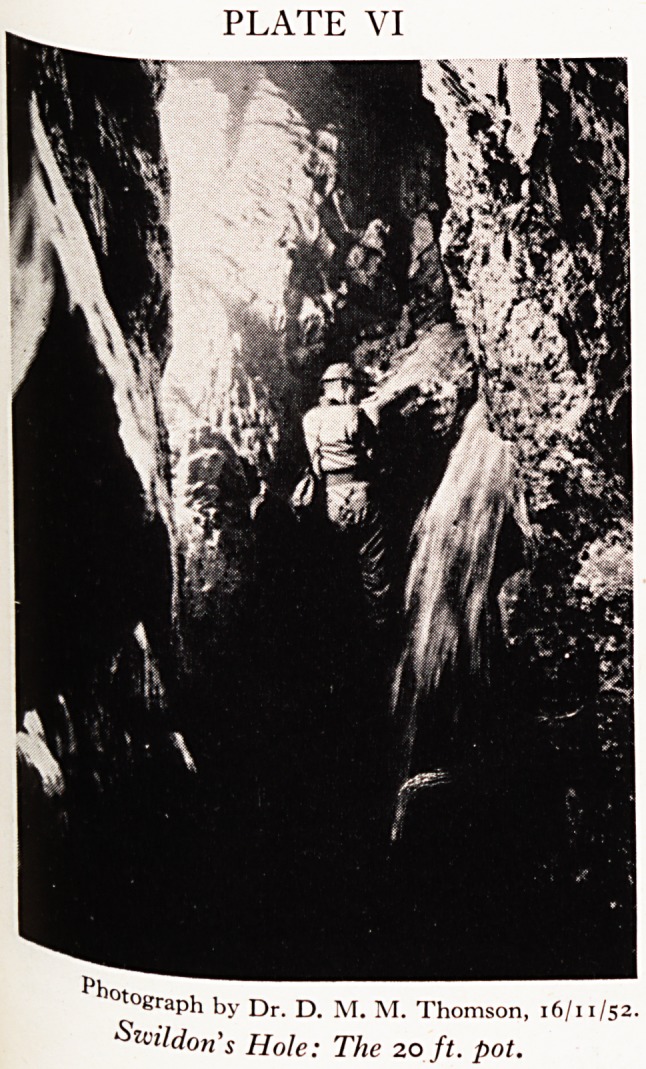


**Figure f5:**
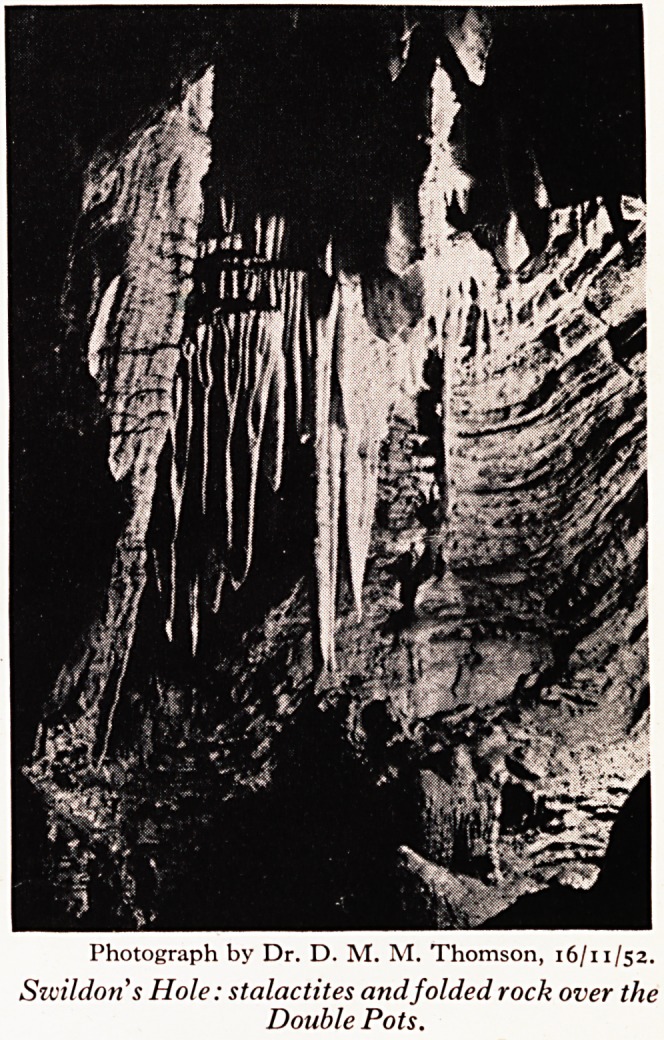


**Figure f6:**
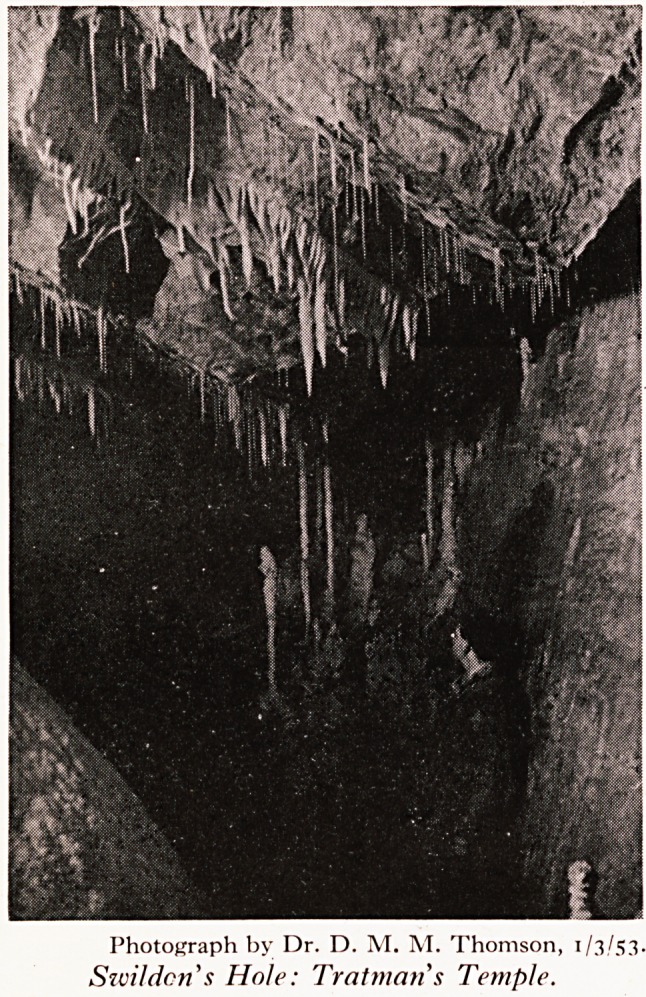


**Figure f7:**
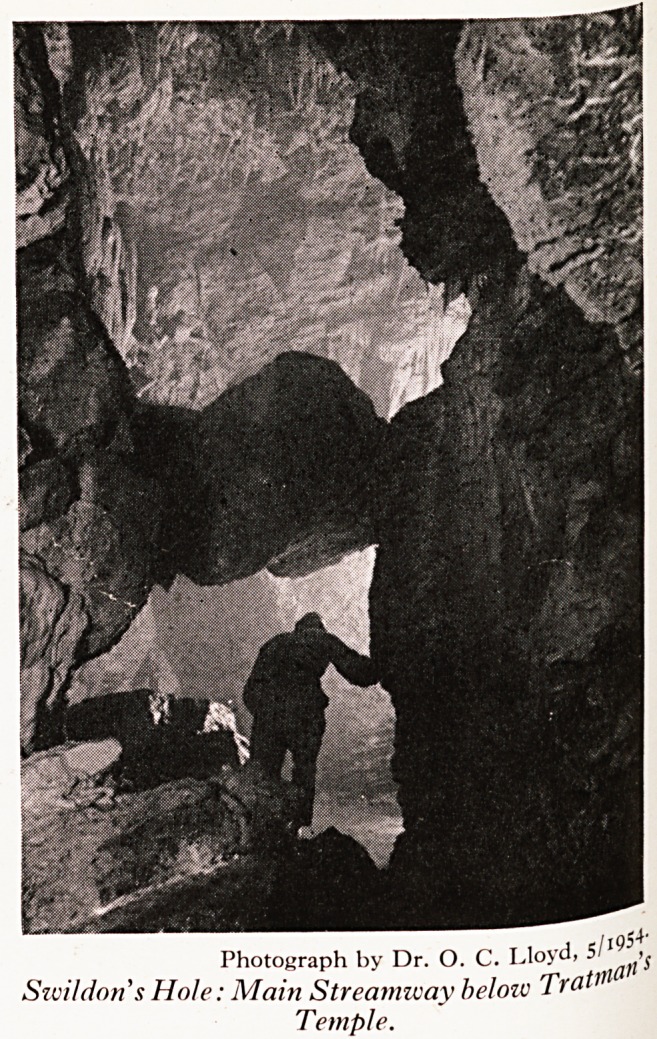


**PLATE X f8:**
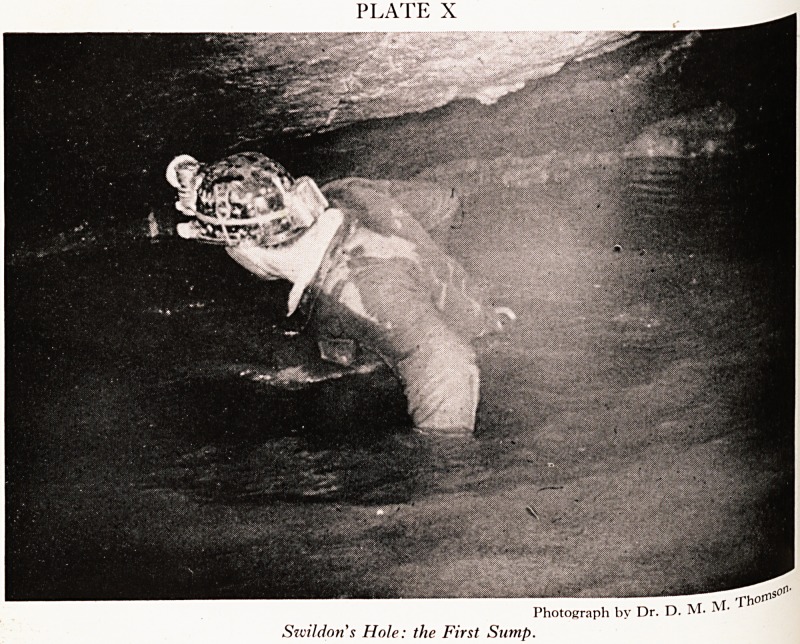


**Figure f9:**
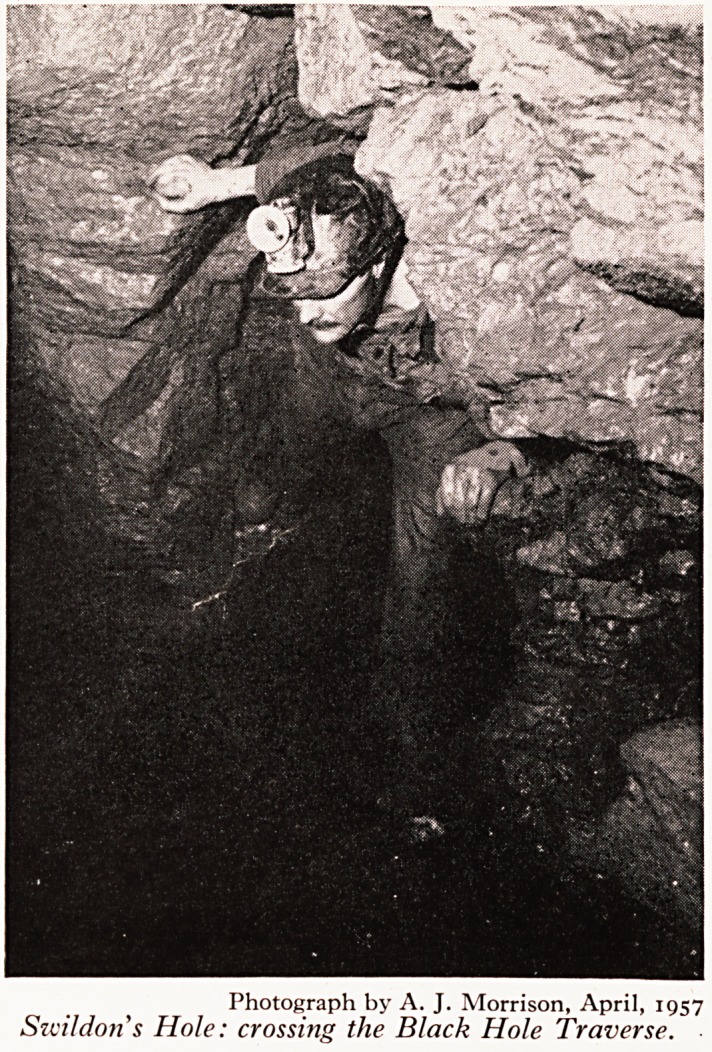


**PLATE XII f10:**
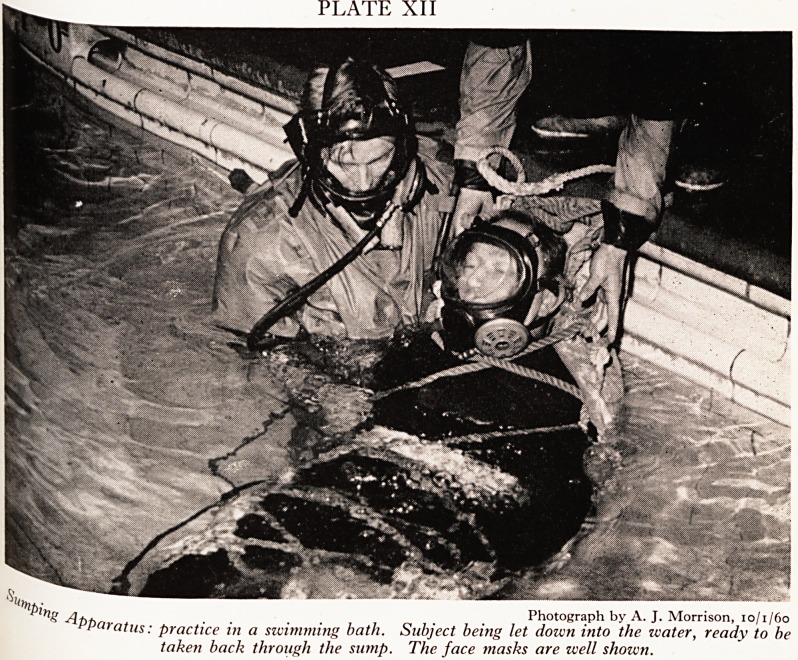


**PLATE XIII f11:**
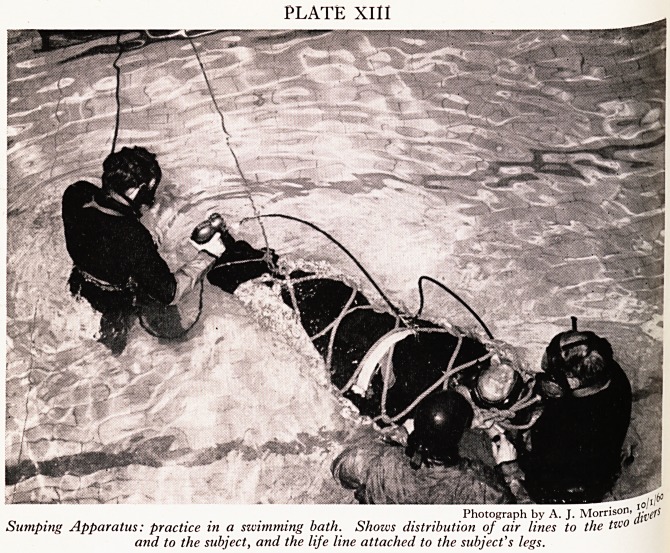


**Figure f12:**
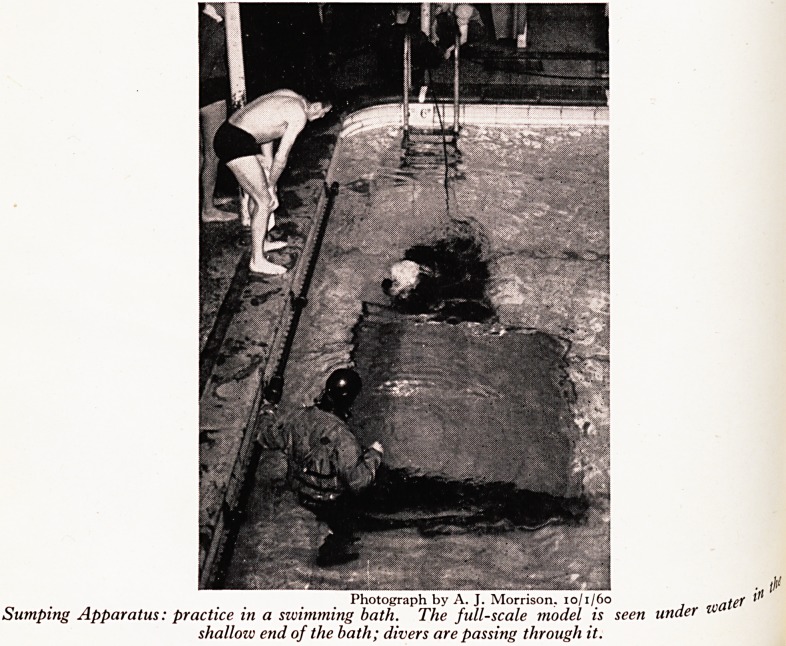


**Fig. 3. f13:**
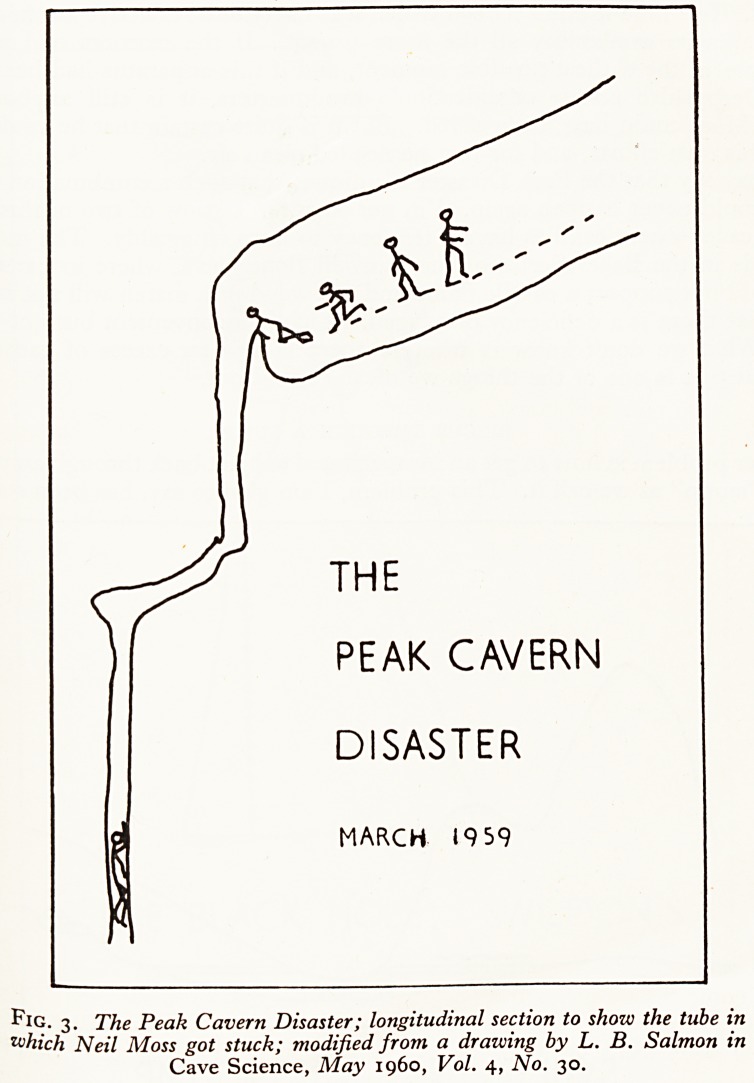


**Fig. 4. f14:**
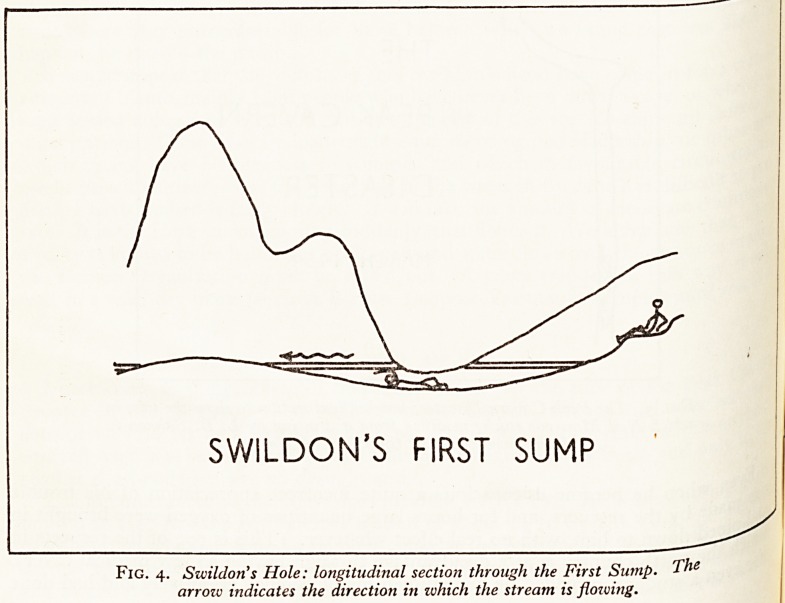


**Fig. 5. f15:**